# Identification of Medically Actionable Secondary Findings in the 1000 Genomes

**DOI:** 10.1371/journal.pone.0135193

**Published:** 2015-09-02

**Authors:** Emily Olfson, Catherine E. Cottrell, Nicholas O. Davidson, Christina A. Gurnett, Jonathan W. Heusel, Nathan O. Stitziel, Li-Shiun Chen, Sarah Hartz, Rakesh Nagarajan, Nancy L. Saccone, Laura J. Bierut

**Affiliations:** 1 Department of Psychiatry, Washington University School of Medicine, St Louis, Missouri, United States of America; 2 Department of Pathology and Immunology, Washington University School of Medicine, St Louis, Missouri, United States of America; 3 Department of Genetics, Washington University School of Medicine, St Louis, Missouri, United States of America; 4 Division of Gastroenterology, Department of Medicine, Washington University School of Medicine, St Louis, Missouri, United States of America; 5 Department of Neurology, Washington University School of Medicine, St Louis, Missouri, United States of America; 6 Cardiovascular Division, Department of Medicine, Washington University School of Medicine, St Louis, Missouri, United States of America; 7 Division of Statistical Genomics, Washington University School of Medicine, St Louis, Missouri, United States of America; 8 Chief Informatics Officer, Pierian DX, St Louis, Missouri, United States of America; University of Southern California, UNITED STATES

## Abstract

The American College of Medical Genetics and Genomics (ACMG) recommends that clinical sequencing laboratories return secondary findings in 56 genes associated with medically actionable conditions. Our goal was to apply a systematic, stringent approach consistent with clinical standards to estimate the prevalence of pathogenic variants associated with such conditions using a diverse sequencing reference sample. Candidate variants in the 56 ACMG genes were selected from Phase 1 of the 1000 Genomes dataset, which contains sequencing information on 1,092 unrelated individuals from across the world. These variants were filtered using the Human Gene Mutation Database (HGMD) Professional version and defined parameters, appraised through literature review, and examined by a clinical laboratory specialist and expert physician. Over 70,000 genetic variants were extracted from the 56 genes, and filtering identified 237 variants annotated as disease causing by HGMD Professional. Literature review and expert evaluation determined that 7 of these variants were pathogenic or likely pathogenic. Furthermore, 5 additional truncating variants not listed as disease causing in HGMD Professional were identified as likely pathogenic. These 12 secondary findings are associated with diseases that could inform medical follow-up, including cancer predisposition syndromes, cardiac conditions, and familial hypercholesterolemia. The majority of the identified medically actionable findings were in individuals from the European (5/379) and Americas (4/181) ancestry groups, with fewer findings in Asian (2/286) and African (1/246) ancestry groups. Our results suggest that medically relevant secondary findings can be identified in approximately 1% (12/1092) of individuals in a diverse reference sample. As clinical sequencing laboratories continue to implement the ACMG recommendations, our results highlight that at least a small number of potentially important secondary findings can be selected for return. Our results also confirm that understudied populations will not reap proportionate benefits of genomic medicine, highlighting the need for continued research efforts on genetic diseases in these populations.

## Introduction

The use of exome and genome sequencing is swiftly increasing in medicine. In addition to identifying specific findings related to the indication for sequencing, these assays that assess a large portion of our genes may uncover other clinically relevant variants. These variants may be deliberately searched for (secondary findings) or accidentally discovered (incidental findings) during the course of sequencing [1]. Though the concept of secondary and incidental findings is not new to medicine [2] or genetics [3], the likelihood of uncovering these findings has dramatically increased with genomic sequencing [4, 5].

In March 2013, the American College of Medical Genetics and Genomics (ACMG) recommended that clinical sequencing laboratories return pathogenic variants in 56 genes associated with 24 medically actionable conditions [6, 7]. These recommendations prompted a heated debate. Critics emphasize the patient’s right to choose to receive these findings and object to a mandatory duty to assess and report results [8–10]. They highlight that the predictive value of disease-associated variants in the general population is unknown, and that variants may be identified at a high frequency, leading to undue anxiety and unnecessary procedures [9, 10]. The ACMG board has subsequently modified its recommendation to include an “opt out” option. Proponents of the recommendations argue that for well-established pathogenic variants associated with the proposed conditions, surveillance and intervention may be lifesaving [11, 12]. Furthermore, similar to other areas of medicine, sequencing laboratories have a responsibility to comprehensively evaluate test results. The ACMG working group acknowledges that there are limited data to fully support their recommendations and advises regular review and update of the list [6, 7].

Uniformly, there is a call for more research on the ACMG recommended genes and conditions in the general population [6, 9–11]. This genetic and ethical landscape motivated us to test a stringent approach for identifying clinically relevant secondary findings associated with the ACMG list in the 1000 Genomes dataset [13], a diverse sequencing reference sample. Our goal was to estimate the likelihood of observing secondary findings with substantial evidence for disease association to provide insight into the potential implications of these controversial recommendations.

## Materials and Methods

Our analysis focused on identifying actionable pathogenic and likely pathogenic variants in the 56 ACMG genes (Table 1). Because prevalence estimates of these conditions range from 1/200 to 1/1,000,000 (S1 Table), the probability of an individual in the 1000 Genomes dataset having one of these conditions is low. Thus, a threshold with high specificity for identifying secondary findings is critical to reduce false positive results that may lead to unnecessary procedures and altered life planning. Our approach emphasizes specificity by integrating informatics filtering, literature review, and expert evaluation.

**Table 1:** Number of candidate variants after different review stages.

				Number of Candidate Variants			
Diseases	MIM Disorder	Genes	MIM Gene	Extracted from 1000 Genomes	After Filtering	After Literature Screening	After Specialist Review
Hereditary breast and ovarian cancer	604370	*BRCA1* *	113705	879	15		
	612555	*BRCA2* *	600185	1093	22	1	1
Li-Fraumeni syndrome	151623	*TP53* *	191170	331	4	1	1
Peutz-Jeghers syndrome	175200	*STK11* *	602216	485	.		
Lynch syndrome	609310	*MLH1* *	120436	923	8	1	
	120435	*MSH2* *	609309	2649	6		
	614350	*MSH6* *	600678	1673	2		
	614337	*PMS2* *	600259	459	.		
Familial adenomatous polyposis	175100	*APC* *	611731	2057	11		
MYH-associated polyposis; adenomas, multiple colorectal FAP type 2; colorectal adenomatous polyposis, autosomal recessive with pilomatricomas	608456, 132600	*MUTYH* *	604933	150	5		
Von Hippel-Lindau disease	193300	*VHL* *	608537	189	1		
Multiple endocrine neoplasia, type 1	131100	*MEN1* *	613733	76	1		
Multiple endocrine neoplasia, type 2	171400, 162300	*RET*	164741	734	6		
Familial medullary thyroid cancer	1552401	*RET*	164761	(above)			
PTEN hamartoma tumor syndrome	153480	*PTEN* *	601728	1250	.		
Retinoblastoma	180200	*RB1* *	614041	2127	3		
Hereditary paraganglioma- pheochromocytoma syndrome	168000 (PGL1)	*SDHD* *	602690	402	.		
	601650 (PGL2)	*SDHAF2*	613019	225	.		
	605373 (PGL3)	*SDHC* *	602413	753	.		
	115310 (PGL4)	*SDHB* *	185470	409	6	1	
Tuberous sclerosis complex	191100	*TSC1* *	605284	680	3		
	613254	*TSC2* *	191092	708	3		
*WT1*-related Wilms tumor	194070	*WT1* *	607102	711	2		
Neurofibromatosis type 2	101100	*NF2* *	607379	1034	2		
Ehlers–Danlos syndrome, vascular type	130050	*COL3A1* *	120180	475	3		
Marfan syndrome, Loeys–Dietz syndromes, and familial thoracic aortic aneurysms and dissections	154700	*FBN1* *	134797	2999	11		
	609192	*TGFBR1* *	190181	629	.		
	608967	*TGFBR2* *	190182	1282	1		
	610168	*SMAD3* *	603109	1836	.		
	610380	*ACTA2* *	102620	728	.		
	613795	*MYLK* *	600922	3650	.		
	611788	*MYH11* *	160745	2521	2		
Hypertrophic cardiomyopathy, dilated cardiomyopathy	115197	*MYBPC3* *	600958	219	7		
	192600	*MYH7*	160760	343	7	1	
	601494	*TNNT2* *	191045	304	.		
	613690	*TNNI3*	191044	106	1		
	115196	*TPM1*	191010	445	.		
	608751	*MYL3*	160790	273	2		
	612098	*ACTC1*	102540	128	.		
	600858	*PRKAG2*	602743	5343	.		
	301500	*GLA* *	300644	94	.		
	608758	*MYL2*	160781	148	.		
	115200	*LMNA* *	150330	591	2		
Catecholaminergic polymorphic ventricular tachycardia	604772	*RYR2*	180902	11765	6	1	1
Arrhythmogenic right-ventricular cardiomyopathy	609040	*PKP2* *	602861	1413	7	1	1
	604400	*DSP* *	125647	637	8		
	610476	*DSC2* *	125645	426	3		
	607450	*TMEM43*	612048	278	1		
	610193	*DSG2* *	125671	660	3		
Romano-Ward Long QT Syndromes Types 1,2, and 3, Brugada Syndrome	192500	*KCNQ1* *	607542	5974	3		
	613688	*KCNH2* *	152427	403	4	1	1
	603830, 601144	*SCN5A* *	600163	1452	18	2	
Familial hypercholesterolemia	143890	*LDLR* *	606945	645	19	5	1
	603776	*APOB*	107730	653	4		
		*PCSK9*	607786	446	3		
Malignant hyperthermia susceptibility	145600	*RYR1*	180901	2335	20		
		*CACNA1S*	114208	1237	2		
Total				70,435	237	15	7

* Genes for which novel, expected pathogenic variants should be returned.

### 1,000 Genomes Sample

Phase 1 of the 1000 Genomes dataset provides low coverage whole-genome sequencing (average 5x) and high coverage exome-sequencing (average 80x) on 1,092 unrelated individuals from 14 different populations in 4 major ancestry groups; Europe, East Asia, Africa, and the Americas [13]. These populations were selected based on scientific, ethical, and practical considerations with the goal of building a resource illustrating the spectrum of geographic genetic variation. Our analysis focused on examining the 56 well-established ACMG genes in the 1,092 individuals in Phase 1 of the 1000 Genomes dataset.

### Ethics Statement

The 1000 Genomes dataset is coded data, which is publically available and unrestricted online through an open access policy. The Washington University Human Research Protection Office determined that this project did not involve activities that were subject to Institutional Review Board oversight.

### Filtering of Variants

**Fig 1:**
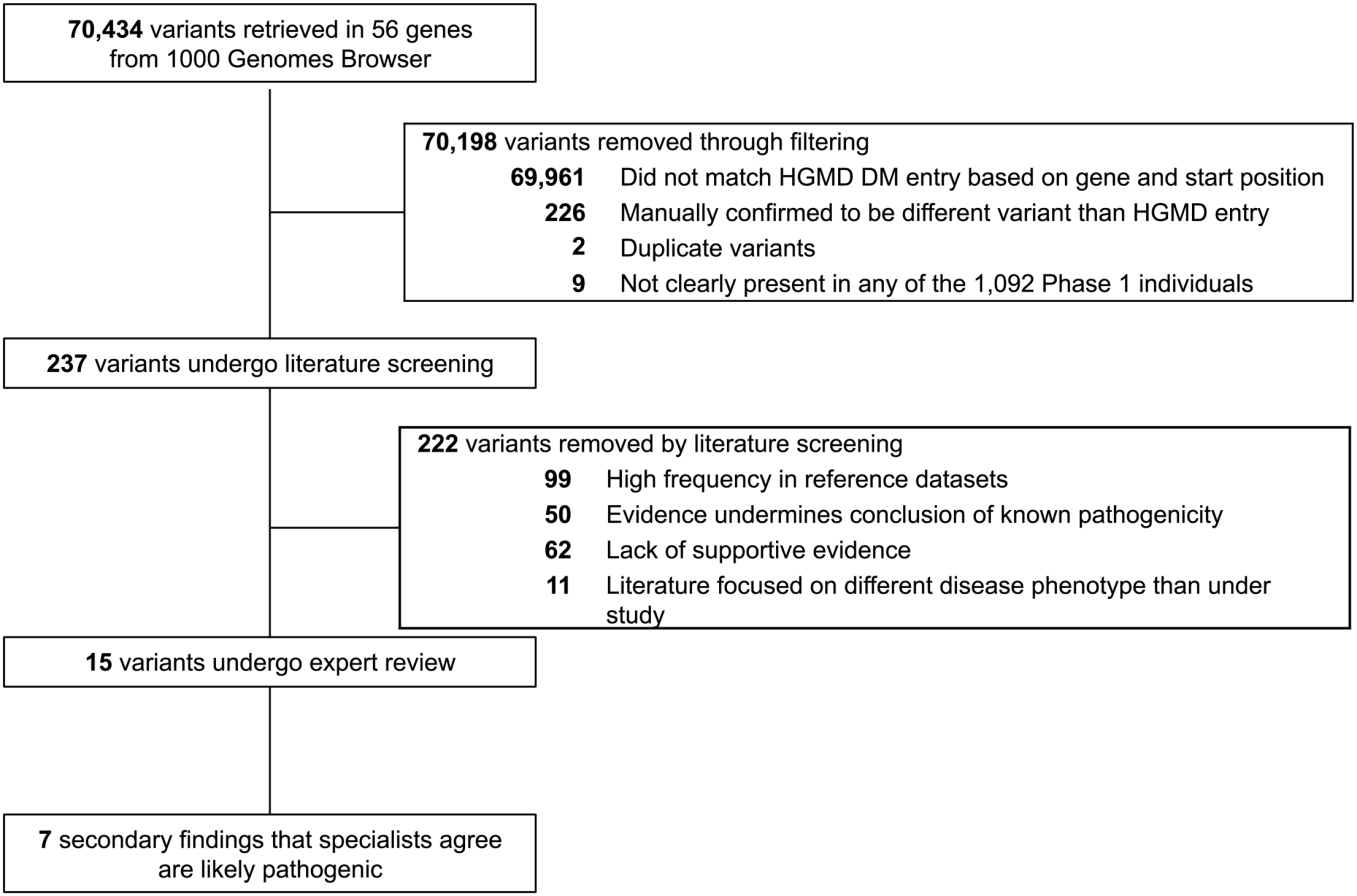
Flow of candidate variants through informatics filtering, literature review, and expert evaluation. Candidate variants in 56 genes associated with 24 actionable conditions from the 1000 Genomes dataset were narrowed down to identify 7 secondary findings that specialists agree are pathogenic or likely pathogenic.

Informatics filtering strategies similar to those proposed by Berg and colleagues [14] narrowed down the number of candidate variants (detailed in Fig 1). Briefly, variants in the 56 genes were downloaded in October 2013 from the 1000 Genomes Browser based on Ensembl version 73 (http://browser.1000genomes.org/index.html). MySQL was used to intersect the downloaded variants with the Human Gene Mutation Database (HGMD) Professional (2.2012) [15]. These variants were filtered by selecting variants labeled disease-causing by HGMD, combining duplicate entries, and eliminating variants retrieved from the 1000 Genomes Browser, but not occurring in the 1,092 Phase 1 individuals.

### Screening of Candidate Variants with Literature Review

Filtered candidate variants were vetted for disease association through critical appraisal of the literature from HGMD Professional [15], ClinVar [16], Google, PubMed, and other relevant databases [17–19]. Variant frequency in the 1000 Genomes and the NHLBI Exome Sequencing Project was also considered with the literature review [20]. Details on all filtered variants along with notes and references from the review are available in S2 Table.

First, variants with an allele frequency greater than expected for the associated disorder in either the NHLBI Exome Sequencing Project (ESP) and/or Phase 1 of the 1000 Genomes were removed. General population disease frequencies were estimated from GeneReviews, the Genetics Home Reference, and the literature review (S1 Table). Similar to Dorschner et al. [21], we assumed that if a variant was found more commonly in reference datasets than expected given the frequency of the associated disease, it is unlikely to cause a high-penetrance phenotype. However, because of the possibility of ancestry-specific disease-causing variants, we used a cautious threshold at this stage. We assumed that the occurrence of multiple variants within each reference dataset followed a Poisson distribution, and specific variants were excluded if the number of occurrences exceeded the 95^th^ cumulative probability percentile with an event rate equal to the expected number of pathogenic variants with the associated disorder (unless this number was 3 or less and then we used a cut off of 4 variants). Although we sought to incorporate information on population-specific frequencies of diseases and variants from the literature, we found that this additional information did not prevent the exclusion of variants using our cautious threshold.

Second, primary literature was evaluated for several lines of evidence against the pathogenicity of each variant to remove false positive results. Variants with similar frequencies in case-control studies, those often seen in healthy individuals, those that did not segregate with the disease in an affected family, those described to coexist with multiple deleterious variants, and those occurring in trans to a single deleterious variant without the expected phenotypic effects of biallelic alteration were removed from consideration. Cancer predisposition variants without loss of heterozygosity in multiple tumors were removed. For *BRCA1* and *BRCA2*, we removed variants with an odds of neutrality greater than 100:1 based on Myriad Genetic Laboratories published data [22], however, the vast majority of Myriad data are not publicly available. For Lynch syndrome variants, we required microsatellite instability within the majority of reported tumors. For variants in *MUTYH* associated with recessive polyposis and colorectal cancer, we excluded those that did not co-occur with another potentially pathogenic mutation as the ACMG guidelines recommend only searching for individuals with biallelic alteration [6].

Third, as we set the threshold for inclusion, we recognized the potential life-changing implications of returning secondary findings, and so we required a minimum level of supportive evidence for non-synonymous, splice site, and synonymous variants to be considered an actionable secondary finding. Similar to the classification system of pathogenic secondary findings employed by Ng et al. [23] and Dorschner et al. [21], we required that the variant was identified in at least three unrelated affected individuals, exhibited segregation consistent with a probability ≤1/16 in at least one family, or occurred in at least one de novo event in a trio. For truncating mutations identified in HGMD Professional that occurred in genes in which the ACMG specified that expected pathogenic variants should be returned (starred in Table 1), we only required a truncating mutation in one unrelated case.

Finally, variants identified in literature focusing on conditions other than the specified ACMG conditions were removed.

### Verification of Pathogenic Variants

Concordance between a clinical laboratory specialist and an expert physician was required to call variants pathogenic or likely pathogenic. All experts were asked to consider the draft “Standards and Guidelines for the Interpretation of Sequence Variants: A Joint Consensus Recommendation of the American College of Medical Genetics and Genomics and the Association of Molecular Pathology” in their evaluation (https://www.acmg.net/docs/Standards_Guidelines_for_the_Interpretation_of_Sequence_Variants.pdf). This consensus statement supports a five tiered variant classification system: 1) pathogenic, 2) likely pathogenic, 3) uncertain significance, 4) likely benign, and 5) benign. Specifically, the consensus statement endorses that “pathogenic” implies causative for disease, and likely pathogenic implies more than 90% certainty that a variant is disease-causing.

A clinical laboratory specialist with board certification in cytogenetics and molecular genetics (CEC) evaluated all remaining variants after literature screening. The clinical laboratory specialist employed genomic browsers including UCSC and Ensembl, genetic databases [18, 19, 24], and protein prediction software [25–27]. This methodology is standard for clinical reporting [28, 29]. Expert physicians with medical specialties relevant to the remaining disease-associated variants also examined the pathogenicity evidence. Specifically, physicians with specialties in gastroenterology (NOD), neurology and pediatrics (CG), pathology (JWH), and cardiovascular medicine (NOS) were provided with the primary literature on variants in their respective fields and asked whether each variant was “actionable” and “pathogenic.”

### Additional Expected Pathogenic Variants

For 45 of the 56 genes (starred in Table 1), the ACMG recommendations suggest that expected pathogenic variants should also be sought and returned to patients. For these 45 genes, we additionally examined variants that were predicted to cause a truncation, but were not listed as disease-causing in HGMD Professional. ANNOVAR was used to examine vcf files, and truncating mutations were identified with refGene and ensGene using Genome Build 19. Identified mutations were required to cause truncation in all listed Ensembl HGVS isoforms. Predicted truncating mutations were then evaluated with literature review and ClinVar. We required that a “pathogenic” truncating mutation had been previously described 3' of the variant under review in the coding sequence for one of the ACMG conditions in either ClinVar or another database, as nonsense mediated decay may not be predicted in transcripts with distal alterations. Expected pathogenic variants were reviewed by the clinical laboratory specialist.

## Results

### Computationally filtered variants

We retrieved 70,435 variants in the 56 disease-associated genes from the 1000 Genomes Browser. After querying HGMD Professional based on gene and chromosome position for variants labeled disease-causing and restricting to variants that matched the exact base change, 237 variants remained for manual review (Fig 1).

Among the 1,092 Phase 1 genomes, our HGMD filtering strategy yielded 1.48 variants per person (Table 2). Across the four major ancestry groups, the average number of variants per person ranged from 1.13 among Asian Americans to 1.67 among the Americas individuals. These findings underscore that filtering using HGMD Professional dramatically reduced the number of candidate secondary variants per genome.

**Table 2:** Distribution of variants per person after filtering, literature screening, and specialist review.

Variants per person	African (n = 246)	the Americas (n = 181)	East Asian (n = 286)	European (n = 379)	Total (n = 1,092)
After filtering	1.463	1.668	1.133	1.667	1.481
After literature screening	0.012	0.011	0.010	0.021	0.015
After specialist review	0.004	0.006	0.003	0.011	0.006

### Literature screened variants

**Fig 2:**
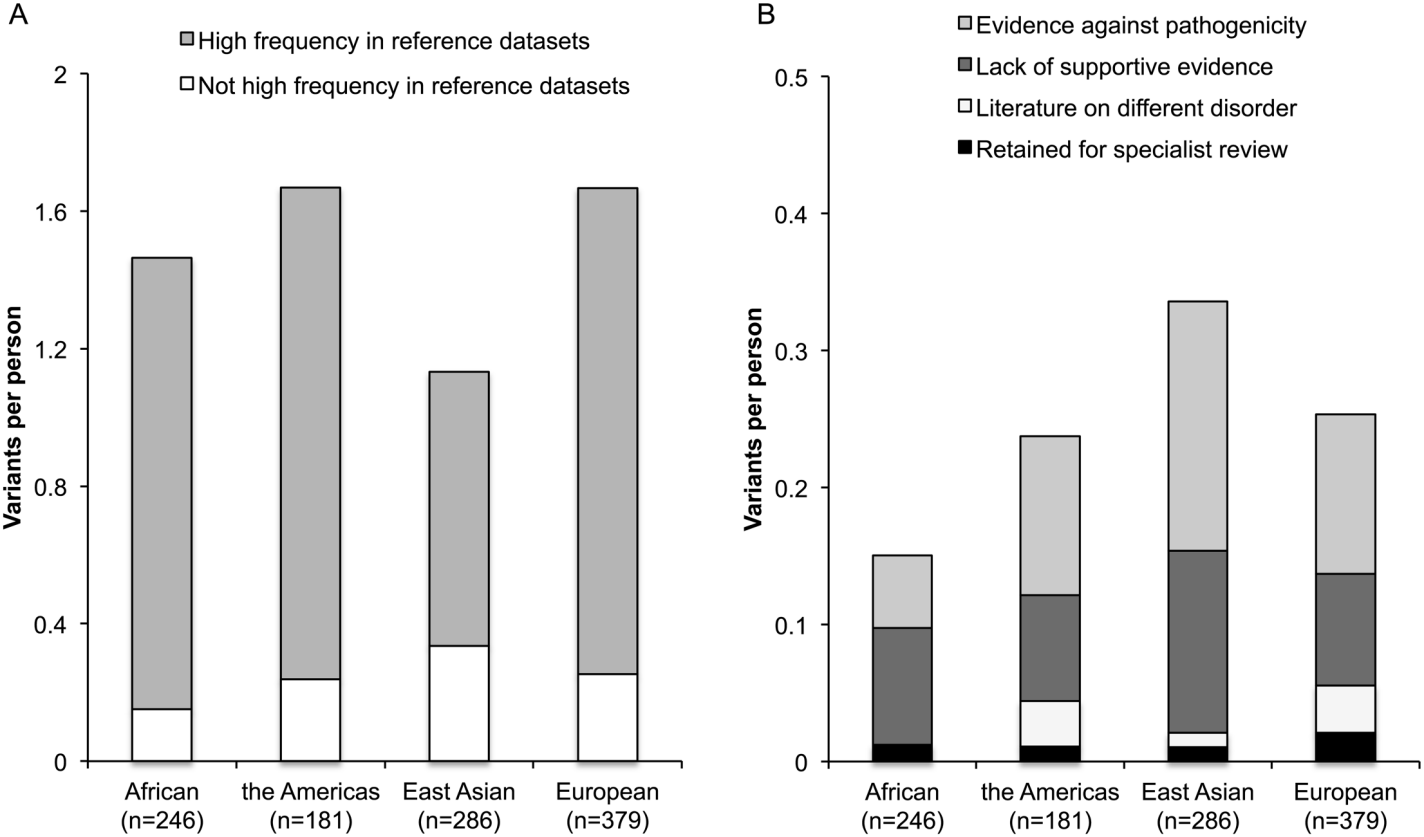
Results of literature screen of 237 filtered candidate variants. These graphs compare the number of variants per person at different stages of the literature screen across the four major ancestry groups in the 1000 Genomes dataset. A) Compares the contribution of variants that were removed because of a high frequency in reference datasets to all of the other filtered variants. B) Compares the contribution to variants per person of all of the filtered variants that did not have a high frequency in reference datasets. Specifically, it compares the contribution of variants with evidence against a conclusion of pathogenicity, a lack of supportive evidence, literature on a different disorder, or those that were retained for specialist review.

Literature appraisal further decreased the number of filtered variants by 15 fold (Table 1, Fig 1). More than one-third of the variants (99/237) were removed because of a higher frequency in reference datasets than expected based on the population prevalence and mode of inheritance of these conditions (details in S1 Table). Fig 2A illustrates that these 99 variants accounted for the majority of variants per person among the 237 filtered candidate variants across the four ancestry groups. Specifically, the number of variants removed per person in this step of the literature screening was 1.43 (86% of total 1.67) in the Americas, 1.41 (85% of total 1.67) in European, 1.31 (90% of total 1.46) in African, and 0.79 (70% of total 1.13) in East Asian ancestry groups.

An additional 50 variants were eliminated because the literature evidence undermined the conclusion of known pathogenicity, including high incidence in healthy individuals, lack of segregation with disease, and co-occurrence with known deleterious variants (Fig 1). Fig 2B illustrates that the number of variants per person removed due to evidence against pathogenicity varied across the ancestry groups. Specifically, the number of variants removed per person in this step of the literature screening was 0.18 (16% of 1.13) in East Asians, 0.12 (7% of 1.67) in Europeans, 0.12 (7% of 1.67) in the Americas, and 0.05 (4% of 1.46) in Africans.

We removed 62 variants that lacked a minimum level of supportive evidence in the literature (Fig 1). Across ancestry groups, the number of variants per person removed due to paucity of evidence was similar (Fig 2B): 0.13 (11% of 1.13) in East Asians, 0.08 (6% of 1.46) in Africans, 0.08 (5% of 1.67) in Europeans, and 0.08 (5% of 1.67) in the Americas. Finally, 11 variants were removed where the literature focused on a different disease phenotype than under study.

Overall, manual literature screening dramatically reduced the number of filtered variants per person from 1.48 to 0.015 (Table 2). After literature screening, 15 variants remained and were reviewed by the clinical laboratory specialist and expert physicians (Fig 1). The specialists independently agreed that 7 of these variants met the high threshold for being pathogenic or likely pathogenic and actionable (Table 3).

**Table 3:** Pathogenic variants that the clinical laboratory specialist and expert physicians agree should be disclosed as secondary findings.

Gene	Genomic change (protein change)	dbSNP Identifier	1000 Genomes	Exome Variant Server	ClinVar	Predictions of Clinical Specialist (CEC)	Predictions of Expert Physicians; Notes (expert initials)
*BRCA2*	Chr13: 32972575G>T (p.Glu3309*)	rs80359251	1 (AFR^a^)	4 (AA)	Pathogenic/ Likely pathogenic	Likely Pathogenic	Pathogenic; Exhibits a functional role in several experiments and characterized as pathogenic by multiple databases (JWH)
*TP53*	Chr17: 7577120G>A (p.Arg273His)	rs28934576	1 (EUR^b^)	0	Pathogenic	Pathogenic	Pathogenic; Second most frequently reported TP53 mutation in COSMIC, and extensive functional support (JWH)
*SDHB*	Chr1: 17359573C>T (p.Arg90*)	rs74315366	1 (EUR)	0	Pathogenic/ Likely pathogenic	Likely pathogenic	Likely pathogenic; Segregates with disease in three small families (CG)
*RYR2*	Chr1: 237608788C>T (p.Arg420Trp)	rs190140598	1 (EUR)	0	Not in database	Likely pathogenic	Likely pathogenic; Multiple probands and biochemical evidence that this is functional; Lacking strong transmission data (NOS)
*PKP2*	Chr12: 32949042G>A^e^	rs111517471	1 (ASN^c^)	0	Pathogenic/ Likely pathogenic	Likely pathogenic	Likely pathogenic; Identified in multiple individuals with some evidence of segregation (NOS)
*KCNH2*	Chr7: 150648826T>C (p.Leu552Ser)	rs199472918	1 (EUR)	0	Pathogenic/ Likely pathogenic	Likely pathogenic	Likely pathogenic; Identified in multiple families and probands, but with incomplete penetrance (NOS)
*LDLR*	Chr19: 11200235G>A (p.Trp4*)	rs201016593	1 (AMR^d^)	0	Not in database	Likely pathogenic	Pathogenic; Expected type of mutation to cause disease, independent reports and biochemical support (NOS)

^*a*^AFR, African.

^*b*^EUR, European.

^*c*^ASN, East Asian.

^*d*^AMR, the Americas.

^*e*^Splice variant.

### Known pathogenic and likely pathogenic variants identified by clinical specialists

A *BRCA2* truncating variant p.Glu3390* occurred in one individual from the 1000 Genomes ASW population (Americans of African Ancestry in SW USA). Previously reported in a case of ovarian cancer, this genetic variant was shown to have a functional effect in a series of biochemical experiments [30]. Based on strong functional support and the nature of the alteration, the clinical laboratory specialist classified this variant as likely pathogenic, and the expert physician (JKH) independently confirmed that the variant was pathogenic for hereditary breast and ovarian cancer.

A *TP53* nonsynonymous variant p.Arg273His was identified in one individual in the CEU population (Utah Residents (CEPH) with Northern and Western European ancestry). Malkin et al. [31] identified this variant in a proband diagnosed with soft-tissue sarcoma and gastric carcinoma as well as in the proband’s son diagnosed with rhabdomyosarcoma at age 11. Fagin et al. [32] found this variant in 5 out of 6 anaplastic thyroid carcinomas. Described as a hotspot mutation, this variant is the second most frequently reported *TP53* mutation in the catalogue of somatic mutations in cancer (COSMIC), and several independent groups have provided functional support. Both the clinical laboratory specialist and expert physician (JKH) thought this variant was pathogenic for Li-Fraumeni syndrome.

A *SDHB* truncating variant p.Arg90* occurred in one individual in the GBR population (British in England and Scotland). Located in a hypermutable CpG dinucleotide, Astuti et al. [33] showed that this variant segregated in 3 unrelated small families suffering from pheochromocytoma and paragangliomas. Based on the literature review and the nature of the alteration, both the clinical laboratory specialist and the expert physician (CG) classified this variant as likely pathogenic.

A *RYR2* nonsynonymous variant p.Arg420Trp occurred in one individual in the CEU population. Bruce et al. [34] identified this variant in two unrelated families in Italy with several cases of juvenile onset cardiac death, but with incomplete penetrance. Because this variant was also identified in several other independent cases and functionally characterized as abnormal, the clinical laboratory specialist and the expert physician (NOS) classified the variant as likely pathogenic for catecholaminergic polymorphic ventricular tachycardia.

A *PKP2* splice region variant c.2489+1G>A occurred in one individual in the CHB population (Han Chinese in Beijing, China). Cox et al. [35] found this variant in 6 unrelated Dutch cases of right ventricular dysplasia/cardiomyopathy. Given that other studies report additional independent cases with some limited transmission data, both the clinical laboratory specialist and the expert physician (NOS) classified the variant as likely pathogenic.

A *KCNH2* nonsynonymous variant p.Leu552Ser was found in an individual from the FIN population (Finnish in Finland). Described as a Finnish founder mutation, this variant was documented by Piippo et al. [36] in 6 unrelated Long QT syndrome Finnish families. Ten of 35 heterozygous individuals were symptomatic (mean QTc of the 35 individuals was 466 ± 47 ms) and all 43 non-carrier family members were non-symptomatic (mean QTc 416 ± 23 ms). Furthermore, two homozygous siblings experienced severe symptoms (2:1 AV block immediately after birth and *torsades de pointes* at age 2). Computational prediction programs further supported this variant’s pathogenicity, and the clinical laboratory specialist and expert physician (NOS) confirmed that it was likely pathogenic.

A *LDLR* truncating variant p.Trp4* was found in one individual from the CLM population (Colombians in Medellin, Colombia). Nonsense variants within *LDLR* codon 4 have been described in a Spanish family, a Chinese individual, and a Colombian individual with familial hypercholesterolemia [37, 38]. Based on literature review and the nature of the alteration, the clinical laboratory specialist classified the variant as likely pathogenic, and the expert physician (NOS) confirmed that the variant was expected to be pathogenic.

Eight of the fifteen variants retained for literature review were determined to be variants of unknown significance by the clinical laboratory specialist (CEC). These classifications were based on several factors, including limited available data, uncertain significance by expert gene curation, occurrence in patients with complex genotypes, and high frequency in reference datasets.

### Additional expected pathogenic variants

Five additional expected pathogenic variants were identified that were not listed as disease-causing in HGMD Professional (Table 4). These truncating variants occur in *BRCA2*, *TGFBR1*, *DSP* (n = 2), and *LDLR*, and ClinVar suggests that mutations located 3’ in the coding sequence of these genes are pathogenic for the ACMG conditions of hereditary breast and ovarian cancer, Loeys-Dietz syndrome type 1A, arrhythmogenic right ventricular cardiomyopathy, and familial hypercholesterolemia, respectively. All of these variants are located within the first 90% of the protein sequence (range of 45%-87%) and therefore are expected to lead to nonsense mediated decay. Due to the nature of these alterations, these variants represent returnable secondary findings according to the ACMG recommendations.

**Table 4:** Additional expected pathogenic variants that meet criteria for disclosure as secondary findings.

Gene	Genomic change (protein change)	dbSNP Identifier	1000 Genomes	Exome Variant Server	Notes from database examination
*BRCA2*	Chr13: 32929053G>T (p.Glu2355*)	rs200078639	1 (AMR^a^)	0	ClinVar: variants later in protein sequence are pathogenic for hereditary breast and ovarian cancer
*TGFBR1*	Chr9: 101900238G>A (p.Trp224*)	rs201021249	1 (EUR^b^)	0	ClinVar: variants later in protein sequence are pathogenic for Loeys-Dietz syndrome type 1A
*DSP*	Chr6: 7583372G>A (p.Trp1959*)	rs201774541	1(ASN^c^)	0	ClinVar: variants later in protein sequence are pathogenic for cardiomyopathy dilated with woolly hair and keraderma or arrhythmogenic right ventricular cardiomyopathy
*DSP*	Chr6: 7584224T>A (p.Tyr2243*)	rs188533371	1 (AMR)	0	ClinVar: variants later in protein sequence are pathogenic for cardiomyopathy dilated with woolly hair and keraderma or arrhythmogenic right ventricular cardiomyopathy
*LDLR*	Chr19: 11233939C>T (p.Arg744*)	rs200793488	1 (AMR)	0	ClinVar: variants later in protein sequence are pathogenic for familial hypercholesterolemia

^*a*^AMR, the Americas.

^*b*^EUR, European.

^*c*^ASN, East Asian.

## Discussion

Our goal was to apply a stringent approach to identify clinically important secondary findings using a diverse reference sample. We focused on the 56 ACMG genes associated with 24 actionable conditions [6]. Our results demonstrate that 12 individuals in Phase 1 of the 1000 Genomes dataset (1%) carry a returnable secondary finding using this standard. The pathogenic and likely pathogenic variants identified here are associated with cancer predisposition syndromes, cardiac conditions, and familial hypercholesterolemia, which are diseases with available, potentially life-saving interventions.

Four individuals were identified in the 1000 Genomes dataset with secondary findings associated with cancer predisposition syndromes (Tables 3 and 4). Likely pathogenic *BRCA2* variants were found in 2 individuals, which is consistent with the estimated general population prevalence of 1/400 of hereditary breast and ovarian cancer syndrome [39]. We also identified one pathogenic variant in *TP53* associated with Li-Fraumeni syndrome, which has an estimated prevalence of 1/5,000-1/20,000 and is characterized by several classic tumors, including soft tissue sarcomas, breast cancer, brain tumors, adrenocortical carcinomas, and leukemias [40]. Finally, one individual had a likely pathogenic variant for hereditary paraganglioma-pheochromocytoma syndrome, a very rare condition, for which early detection through surveillance and removal of tumors may minimize complications related to mass effects, catecholamine hypersecretion, and malignant transformation [41].

Beyond cancer predisposition syndromes, we identified 6 individuals with secondary findings associated with cardiac conditions. Given that these diseases may first present with sudden death, early surveillance and intervention are critical. First, one individual in the 1000 Genomes possessed a truncating variant predicted to cause Loeys-Dietz syndrome type 1A, a connective tissue disorder associated with vascular abnormalities (increased risk of arterial aneurysms and dissections) along with skeletal manifestations [42]. Second, three individuals in the 1000 Genomes had likely pathogenic variants associated with Arrhythmogenic Right-Ventricular Cardiomyopathy (ARVC). Although ARVC has an estimated prevalence of 1/1,000-1/1,500, it often exhibits reduced penetrance (with estimates as low as 20–30%), possibly explaining our recognition of 3 disease-associated variants in the 1000 Genomes dataset [43, 44]. Characterized by progressive fibrofatty replacement of the myocardium, ARVC predisposes individuals to ventricular tachycardia and sudden death. Third, one individual in the 1000 Genomes had a likely pathogenic variant associated with catecholaminergic polymorphic ventricular tachycardia (CPVT), which has an estimated prevalence of 1/10,000 and is characterized by episodes of ventricular tachycardia often triggered by exercise, possibly leading to ventricular fibrillation and sudden-death [45]. Finally, we identified one individual with a secondary finding for long QT syndrome, which has an estimated prevalence of 1/2,500 among whites [46] and is characterized by QT prolongation and T-wave abnormalities on ECG with risk of *torsades de pointes* [47].

Lastly, two individuals had likely pathogenic truncating variants in *LDLR* associated with heterozygous familial hypercholesterolemia, which is consistent with the estimated population prevalence of 1/200-1/500 [48]. Characterized by elevated LDL cholesterol levels from birth, this condition increases risk of premature coronary heart disease. Early diagnosis and treatment with statins can decrease coronary heart disease events and mortality [49, 50].

Overall, this study identifies 12 pathogenic and likely pathogenic variants in the 1000 Genomes dataset, which if recognized and returned could guide medical follow-up for individuals and their families. This confirms that medically relevant secondary findings can be identified in an unselected cohort.

Beyond assessing the general frequency of secondary findings, this study provides insight into the frequency of candidate variants in a range of populations. After computational filtering, the average number of variants per person ranged from 1.67 among Europeans to 1.13 among East Asians (Table 2). After literature and expert review, 4 of the 7 identified known secondary findings were observed in individuals of European ancestry, and 1 was found in each of the other ethnic groups (African, the Americas, and East Asian) (Table 3). Examination of secondary findings in the Exome Sequencing Project also identified these findings in European Americans at over three times the rate as African Americans [21, 51]. These observations reflect the historical focus of clinical genetic research on individuals of European descent. We found that a disproportionately low number of individuals of East Asian ancestry had variants that were ruled out due to high frequency in reference datasets, reflecting the fact that one of the two reference datasets was the Exome Sequencing Project, which only contains European and African Americans. Because African Americans have not been well-studied in the literature, we also observed that a lower number of individuals in this group had variants that were ruled out because of evidence against pathogenicity. As return of secondary and incidental findings expands in response to the recent ACMG recommendations [6], understudied populations will not reap proportionate benefits and disparities can increase, highlighting the need for research on genetic diseases in these populations.

Previous reports have predicted substantially higher frequencies of pathogenic variants in the 1000 Genomes dataset. Surveys based on the pilot of the 1000 Genomes project found that each genome typically contains 100 loss-of-function variants [52] and 40–110 variants classified by HGMD Professional as disease-causing (of which 0–8 are predicted to be highly damaging) [53]. A study of the 1,092 Phase 1 genomes found on average 294 previously identified pathogenic variants in the homozygous state in each individual using HGMD [54]. More recently, Daneshjou et al. [55] examined the 1,092 Phase 1 genomes along with 178 additional genomes and found that, after excluding the most common variant, 20% of all analyzed genomes possessed designated ClinVar pathogenic variants in the ACMG genes. Our estimate is considerably lower because we employed a purposefully stringent approach for prioritizing clinically meaningful findings that involved manual curation.

Studies that employ informatics filtering and strict manual review support our observation that a small number of variants for actionable conditions can be prioritized. Johnston et al. [56] employed filtering and manual review to assess 37 genes associated with cancer predisposition syndromes in 572 predominantly white ClinSeq research participants, identifying 8 individuals with pathogenic variants that warranted follow-up. Ng et al. [23] examined 870 ClinSeq research participants for 63 genes associated with cardiomyopathies and arrhythmias and identified 6 individuals with pathogenic variants. More recently, Amendola et al. [51] examined 112 actionable genes in the 6,503 participants enrolled in the National Heart, Lung, and Blood Institute Sequencing Project, identifying 113 individuals with pathogenic, likely pathogenic, or expected pathogenic variants. Proportionate to the number of genes studied, these estimates of 0.014 [56], 0.007 [23], and 0.017 [21] secondary findings per person are on the same order of magnitude as our estimate of 0.011. These estimates from independent samples indicate that a small number of disease-associated variants can be selected from sequence data.

An important limitation of this study is that the informed consent process for the 1000 Genomes project prevents the return of individual research results [57]. This inability to return results with potentially lifesaving interventions underscores a drawback of studies that stress collection of de-identified samples. In designing future genetic studies, including the recent Precision Medicine Initiative [58], investigators need to consider offering a path for returning medically important results identified through the research process to participants. In many surveys, the public strongly favors opportunities to receive individual genetic research results [59, 60]. In addition, return of results is necessary to understand the penetrance and expressivity of the identified secondary findings through medical follow-up. As emphasized by the ACMG [6] and others [9, 10], more research on the long term phenotypic effects of presumed pathogenic variants identified in the general population is needed to fully understand the costs and benefits of returning secondary findings.

There are also several limitations to our method of variant prioritization that may miss pathogenic variants. First, the limited set of 56 ACMG genes was assessed. Inclusion of additional conditions will increase the frequency of actionable secondary findings. Second, filtering based on HGMD Professional entries may exclude expected pathogenic variants that have not been annotated as disease-causing in this database. A third limitation is the reliance on supporting publications to assess pathogenicity given that publications have predominantly focused on European ancestry populations. Efforts to share information in the genetics community through centralized databases [61] will improve the fund of knowledge on genetic variants and provide additional information needed to assess very rare variants. All of these limitations underestimate the frequency of secondary findings, consistent with our stringent approach for variant prioritization. This study is a systematic attempt to combine available information to identify clinically relevant secondary findings, and this framework can be modified as knowledge of genetic diseases increases and guidelines regarding return of secondary and incidental findings continue to evolve.

Our experience of evaluating secondary findings highlights some of the current challenges faced by clinical laboratories in implementing the ACMG recommendations. Although HGMD Professional [15] is useful for filtering candidate variants (Fig 1), our results confirm previous reports that it contains variants designated as disease-causing that upon further review have uncertain pathogenicity for the purposes of secondary finding identification [14, 21, 56]. Our process of secondary finding evaluation required several steps of time-consuming manual review. Informatics filtering led to 237 HGMD disease-causing variants, which each underwent literature screening, requiring approximately 1.5 hours per variant (range of 0.5 to 3 hours). Fifteen candidate variants passed literature review and were evaluated by both a clinical laboratory specialist and an expert physician. Expert review took approximately 1 hour per variant for each specialist (range of 20 minutes to 4 hours). From the time that this project was initiated in 2013, the speed of variant review has dramatically improved with the development of new appraisal resources and additional experience of the authors in variant evaluation. Future efforts to develop standardized resources with well-curated variants to facilitate the fast and accurate identification of pathogenic secondary findings that meet current standards for return in clinical settings will make the implementation of precision medicine more efficient.

Variant appraisal is also complicated by the different thresholds specialists have for identifying pathogenic variants. Our method used a conservative clinical approach by requiring that both a clinical laboratory specialist and expert physician independently agreed that the secondary findings were pathogenic/likely pathogenic. Although initially there was some discordance in classification between the experts, further discussion with the expert reviewers led to agreement for all candidate variants that passed the literature screen. The ACMG [28] and others[29] have released standardized guidelines for variant evaluation that will aid specialists in assessing pathogenic variants (see https://www.acmg.net/ACMG/Publications/Laboratory_Standards___Guidelines/ACMG/Publications/Laboratory_Standards___Guidelines.aspx?hkey=8d2a38c5-97f9-4c3e-9f41-38ee683bcc84). Our experience illustrates that differences in manual curators can lead to differences in variant categorization, highlighting the importance of continued efforts to specify how specialists should combine data from multiple sources to accurately and reliably identify secondary findings.

## Conclusions

In summary, this study of the 1000 Genomes, a diverse cohort of unselected individuals, demonstrates that a stringent approach can prioritize a small number of secondary findings for which the potential clinical benefits of return are great. This work suggests that following ACMG recommendations using a high threshold for pathogenicity will yield at least a small number of clinically relevant findings. This work has implications for future research studies, including the newly proposed Precision Medicine Initiative that is projected to have over 1 million participants [58]. An extrapolation of our findings indicates that at least 1,000 participants in the Precision Medicine Initiative will have a clinically important secondary finding. Genetic research studies will need to address the ethical and practical issues regarding the return of these medically actionable results. Future efforts to improve methods for the fast and accurate identification of secondary findings are needed to speed the translation of genomics into clinical care.

## Supporting Information

S1 Table General population prevalence estimates of the ACMG conditions and the development of frequency thresholds in reference datasets. Population prevalence estimates of the ACMG conditions were taken from several datasets, including GeneReviews and the Genetics Home Reference. Based on the lowest estimated general population disease prevalence and the mode of inheritance, we calculated the maximum estimated pathogenic variants per person for each disease. From this “pathogenic variants per person” estimate, we were able to calculate an expected number of pathogenic variants for each disease in the NHLBI Exome Sequencing Project and the 1000 Genomes. Assuming that the occurrence of multiple variants within each reference dataset followed a Poisson distribution, we calculated a threshold number of variants that exceeds the 95^th^ cumulative probability percentile with an event rate equal to the expected number of pathogenic variants in that dataset. Keeping with our cautious approach, we removed variants associated with each disease that occurred more frequently than this upper bound 95th percentile in each dataset. Details on all filtered variants along with notes and references of the literature review are available in S2 Table. *When the number of expected people exceeding the 95th cumulative probability percentile was small (3 or less), we used a minimum cut off of 4 individuals to prevent the removal of possible population specific variants.
(DOCX)

S2 Table Characteristics of 237 Filtered Variants from Literature Review. Candidate variants identified by informatics filtering were examined for disease association through critical appraisal of the literature. Variant frequency in reference datasets was considered with the literature review. This table provides detailed information on all 237 variants, including notes from that literature review and PubMed Identification numbers of all articles examined.
(XLS)
