# Supplementary material for: Identification of Medically Actionable Secondary Findings in the 1000 Genomes
Source: PLoS One. 2015 Sep 2;10(9):e0135193. doi: 10.1371/journal.pone.0135193 (PMC4558085; doi:10.1371/journal.pone.0135193)
Supplement: S1 Table — Population prevalence estimates of the ACMG conditions were taken from several datasets, including GeneReviews and the Genetics Home Reference. Based on the lowest estimated general population disease prevalence and the mode of inheritance, we calculated the maximum estimated pathogenic variants per person for each disease. From this “pathogenic variants per person” estimate, we were able to calculate an expected number of pathogenic variants for each disease in the NHLBI Exome Sequencing Project and the 1000 Genomes. Assuming that the occurrence of multiple variants within each reference dataset followed a Poisson distribution, we calculated a threshold number of variants that exceeds the 95th cumulative probability percentile with an event rate equal to the expected number of pathogenic variants in that dataset. Keeping with our cautious approach, we removed variants associated with each disease that occurred more frequently than this upper bound 95th percentile in each dataset. Details on all filtered variants along with notes and references of the literature review are available in S2 Table. *When the number of expected people exceeding the 95th cumulative probability percentile was small (3 or less), we used a minimum cut off of 4 individuals to prevent the removal of possible population specific variants. (DOCX) [file pone.0135193.s001.docx]

**S1 Table 1**.

| **Diseases** | **Range of general population prevalence estimates** | **Maximum estimated pathogenic variants per person** | **Exome Sequencing Project (n=6,501)** | | **1000 Genomes**  **(n=1,092)** | |
| --- | --- | --- | --- | --- | --- | --- |
|  |  |  | Predicted number of pathogenic variants (event rate) | Number of variants exceeding the 95th cumulative probability percentile | Predicted number of pathogenic variants (event rate) | Number of variants exceeding the 95th cumulative probability percentile |
| *BRCA1* and *BRCA2* Hereditary Breast and Ovarian Cancer | 1:400-1:800 | 0.00125 | 16.25 | 23 | 2.73 | 6 |
| Li-Fraumeni Syndrome | 1:5,000-1:20,000 | 0.00010 | 1.30 | 3* | 0.22 | 1* |
| Peutz-Jeghers syndrome | 1:25,000-1:300,000 | 0.00002 | 0.26 | 1* | 0.04 | 1* |
| Lynch Syndrome | 1:440 | 0.00114 | 14.78 | 21 | 2.48 | 5 |
| Familial adenomatous polyposis; APC-Associated Polyposis Conditions | 1:7,000-1:44,000 | 0.00007 | 0.93 | 3 | 0.16 | 1* |
| MUTYH-associated polyposis | 1:20,000-1:40,000 | 0.01414 | 91.94 | 108 | 15.44 | 22 |
| Von Hippel-Lindau Disease | 1:36,000 | 0.00001 | 0.18 | 1* | 0.03 | 1* |
| Multiple Endocrine Neoplasia Type 1 | 1:30,000 | 0.00002 | 0.22 | 1* | 0.04 | 1* |
| Multiple Endocrine Neoplasia Type 2 | 1:35,000 | 0.00001 | 0.19 | 1* | 0.03 | 1* |
| *PTEN* Hamartoma Tumor Syndrome | 1:200,000 | 0.00000 | 0.03 | 1* | 0.01 | 1* |
| Retinoblastoma | 1:15,000-1:20,000 | 0.00003 | 0.43 | 2* | 0.07 | 1* |
| Hereditary paraganglioma- pheochromocytoma syndrome | 1:1,000,000 | 0.00000 | 0.01 | 1* | 0.00 | 1* |
| Tuberous sclerosis complex | 1:5,800-1:6,000 | 0.00009 | 1.12 | 3* | 0.19 | 1* |
| *WT1*-related Wilms tumor | 1:8,000-1:10,000 | 0.00000 | 0.01 | 1* | 0.00 | 1* |
| Neurofibromatosis type 2 | 1:60,000 | 0.00001 | 0.11 | 1* | 0.02 | 1* |
| Ehlers-Danlos syndrome, vascular type | 1:50,000-1:200,000 | 0.00001 | 0.13 | 1* | 0.02 | 1* |
| Marfan syndrome | 1:5,000-1:10,000 | 0.00010 | 1.30 | 3* | 0.22 | 1* |
| Hypertrophic cardiomyopathy | 1:500 | 0.00100 | 13.00 | 19 | 2.18 | 5 |
| Catecholaminergic polymorphic ventricular tachycardia | 1:10,000 | 0.00005 | 0.65 | 2* | 0.11 | 1* |
| Arrhythmogenic right-ventricular cardiomyopathy | 1:1000-1:1250 | 0.00050 | 6.50 | 11 | 1.09 | 3* |
| Long QT syndrome; Romano-Ward Syndrome | 1:3000-1:7000 | 0.00017 | 2.17 | 5 | 0.36 | 2* |
| Familial hypercholesterolemia | 1:200-1:500 | 0.00250 | 32.51 | 42 | 5.46 | 10 |
| Malignant hyperthermia susceptibility | 1:3000-1:70000 | 0.00017 | 2.17 | 5 | 0.36 | 2* |
